# *Penicillium fusisporum* and *P. zhuangii*, Two New Monoverticillate Species with Apical-Swelling Stipes of Section *Aspergilloides* Isolated from Plant Leaves in China

**DOI:** 10.1371/journal.pone.0101454

**Published:** 2014-07-02

**Authors:** Bo Wang, Yun Yu, Long Wang

**Affiliations:** 1 College of Forestry, Northwest A&F University, Yangling, Shaanxi, China; 2 College of Life Sciences, Yangtze University, Jingzhou, Hubei, China; 3 State Key Laboratory of Mycology, Institute of Microbiology, Chinese Academy of Sciences, Beijing, China; University of Brighton, United Kingdom

## Abstract

Two new *Penicillium* species isolated from plant leaves are reported here, namely, *P. fusisporum* (type strain AS3.15338^T^ = NRRL 62805^T^ = CBS 137463^T^) and *P. zhuangii* (type strain AS3.15341^T^ = NRRL 62806^T^ = CBS 137464^T^). *P. fusisporum* is characterized by fast growth rate, apical-swelling monoverticillate penicilli, verrucose stipes, fusiform to oblong conidia about 3.5–4×2–2.5 µm and cinnamon-colored sclerotia. While *P. zhuangii* presents a moderate growth rate, it also bears apical-swelling monoverticillate penicilli but its stipes are smooth-walled, and produces ovoid to globose smooth-walled conidia about 3–3.5 µm. Both species belong to section *Aspergilloides*, and *P. fusisporum* is related to “*P. thomii* var. *flavescens*”, while *P. zhuangii* is morphologically similar to *P. lividum*. Phylogenetic analyses of sequences of calmodulin and beta-tubulin genes both show that the two new taxa form distinct monophyletic clades.

## Introduction

Species of *Penicillium* Link with vesiculate or apical-swelling monoverticillate conidiophores and moderate to fast growth rate were accommodated in the *P. thomii* series, *P. frequentans* series and *P. lividum* series respectively by Raper and Thom [1]. Pitt [2] included 10 species sharing these characters in series *Glabra* Pitt of Subgen. *Aspergilloides* Pitt and regarded *P. glabrum* as the earlier valid name for the species commonly known as *P. frequentans* Westling. Moreover, he broadened the concept of *P. thomii* to encompass 6 synoyms, which was followed by Pitt *et al.* [3]. Based on ITS1-5.8S-ITS2 and LSU ribosomal DNA (rDNA) sequences, Peterson [4] included 7 members of this series in his Goup 2. Later, Barreto *et al.* [5] indicated the heterogeneity of the prevailing species concept of *P. glabrum* and added a new member, *P. subericola* to series *Glabra*. Then, Houbraken and Samson [6] established section *Aspergilloides* Houbraken and Samson based on 4 gene loci to encompass 19 species showing those characters, while other species in Pitt's subgenus *Aspergilloides* were put in sections *Charlesii* and *Sclerotiora*.

In an investigation of phylloplane molds in China, we found many isolates superficially similar to *P. glabrum*, *P. lividum* and *P. thomii*, and thus report here on two new species, *P. fusisporum* sp. nov. and *P. zhuangii* sp. nov. belonging to section *Aspergilloides*.

## Materials and Methods

### Isolation of strains

Leaf samples were collected from trees and kept in sterilized plastic bags. Isolation of phylloplane fungi followed the methods of Nakase and Takashima [7]. No specific permissions were required for these locations/activities. The field studies did not involve endangered or protected species and the GPS coordinates of the specific locations in our study are 31°45′35″N 110°38′57″E, 29°54′43″N 93°10′43″E and 33°52′54″N 107°47′51″E. Eleven strains with vesciculate or apical-swelling stipes and monoverticillate penicilli were obtained and deposited at the China General Microbiological Culture Collection (CGMCC) of Institute of Microbiology, Chinese Academy of Sciences, Beijing, China. The ex-types culture *P. fusisporum* AS3.15338^T^ and *P. zhuangii* AS3.15341^T^ were also deposited at the USDA ARS Culture Collection as NRRL 62805^T^ and NRRL 62806^T^, CBS-KNAW of The Netherlands as CBS 137463^T^ and CBS 137464^T^, respectively. All cultures are also maintained at the corresponding author's laboratory and will be supplied upon request for educational or scientific purpose.

### Morphological studies

Colony characters were assessed using Czapek agar (Cz, Raper and Thom [1], Czapek yeast autolysate agar (CYA, Pitt [2]), 2% malt extract agar (MEA, malt extract (Difco), Pitt [2), YES (yeast extract sucrose agar, yeast extract (Oxoid), Frisvad and Samson [8], and 25% glycerol nitrate agar (G25N, Pitt [2]). Color names followed Ridgway [9]. Wet mounts were prepared using material from colonies growing on MEA at 25°C after 7 d and mounting in 85% lactic acid without dye. Microscopic examination and photography were performed with a Nikon Eclipse 80i microscope equipped with a Nikon DS-L1 Digital Sight system.

### Molecular studies

DNA extraction followed the method of Scott *et al.* [10]. Partial β-tubulin gene (*BenA*) sequences were amplified using the sense primers I2 [11] or Bt2a, with the antisense primer Bt2b [12]; the ITS1-5.8S-ITS2 region of rDNA was amplified using the primers ITS5 and ITS4 [13]; the calmodulin gene (*CaM*) was amplified using the primers of Wang [14]. Polymerase chain reactions (PCR) were carried out in 20 µL reaction mixture containing 0.5 µL of each primer (10 pM/µL), 1.0 µL of genomic DNA (10 ng/µL), 8 µL of 2×PCR MasterMix buffer (0.05 u/µL Taq polymerase, 4 mM MgCl_2_, 0.4 mM dNTPs), and 10 µL of ultra pure sterile water (Biomed Co. Ltd, Beijng, China). Amplifications were performed in a PTC-150 thermocycler (MJ Research, Watertown, Massachusetts, USA), which was programmed for touch-down PCR (TD PCR) consisting of 94°C for 3 min; 94°C for 30 s, 50°C for 30 s, −0.5°C/cycle, 72°C for 45 s, 20 cycles; 72°C for 5 min; 94°C for 30 s, 40°C for 30 s, 72°C for 45 s, 15 cycles; 72°C for 5 min. After amplification the PCR fragments were electrophoresed in 2.0% agarose gels with a 100 bp DNA ladder (MBI Fermentas) at 80 V for 20 min. Gels were then stained in an aqueous 0.5 µg/mL ethidium bromide water solution for 15 min and examined under 254 nm UV using a portable UV light. Samples showing one single, obvious band of the anticipated length on the gel were then purified and sequenced on both strands with an ABI 3700 DNA analyzer (Tsingke Biotechnologies Co., Ltd., Beijing, China). Raw sequences were proof-read and edited manually with BioEdit 7.0.9 [15]. Edited sequences were aligned using muscle in MEGA version 5 [16]. A sum of 43 strains from section *Aspergilloides* (Table 1) with validly published sequences from others' work were included. The *ITS1-5.8S-ITS2* sequences of *P. odoratum* NBRC 7741^T^, “*P. trzebinskianum*” Abe (*nom. inval.*, Art. 36) NBRC 6038^T^ and *P. trzebinskii* NBRC 6110^T^ were retrieved from the on-line catalogue of Biological Resource Center (NBRC), NITE of Japan (http://www.nbrc.nite.go.jp/e/), and the *ITS1-5.8S-ITS2* sequence of *P. trzebinskii* CBS 382.48^T^( = NBRC 6110^T^) can also be downloaded from the Global Mirror System of DNA Barcode Data (GMS-DBD) (http://nz.boldmirror.net/) as Sample ID KAS3070, Sequence ID PATE046-08. The three sequence matrices of the three loci were analyzed using Maximum Likelihood (ML) method and subjected to 1000 bootstrap replications with Kimura-2 parameter model for *CaM* and *BenA* data and General Time Reversible model for *ITS1-5.8S-ITS2* datum, and gaps were treated as partial deletion according to Hall [17]. The aligned sequences (Data S1–3) were submitted to TreeBase (http://purl.org/phylo/treebase/phylows/study/TB2:S14916).

**Table 1:** Forty-four strains included in phylogenetic analyses and the GenBank accession numbers for three genetic markers.

Species	Strains*	Source	Genetic markers#		
			*CaM*	*BenA*	*ITS*
*Penicillium adametzii* K. M. Zaleskii	AS3.4470^T^ = CBS 209.28 ^T^ = NRRL 737^T^	Soil under conifers, Poland	AY678540	JN625957	AF033401
*P.adametzioides* Abe ex G. Smith	CBS 313.59^T^	Unknown source, Japan	JN686387	JN799642	JN686433
*P. angulare* S. W. Peterson, E. M. Bayer & D. T. Wicklow	CBS 130293^T^	Mount Wheeler Road, Red River, New Mexico, USA	KC773804	KC773779	KC773828
*P. bilaiae* Chalab.	NRRL 3391^T^	Soil, Kiev, Ukraine	JN626009	JN625966	AF033402
*P. charlesii* G. Smith	NRRL 778^T^	Moldy corn (*Zea mays*), Italy	AY741754	JX091508	AY742708
*P. expansum* Link	CBS 325.48^T^ = NRRL 976^T^	Apple fruit, USA	DQ911134	AY674400	FJ463031
*P. fellutanum* Biourge	NRRL 746^T^	Unknown source, USA	AY741753	EF198545 (NRRL 35619)	AF033399
*P. fuscum* (Sopp) Biourge	CBS 295.62^T^ = NRRL 3008^T^	Soil, conifer and hardwood forest, Wisconsin, USA	GQ367539	GQ367513	AF033411
***P. fusisporum*** ** L. Wang, sp. nov.**	**AS3.15338^T^**	**Leaves of ** ***Rhododendron*** ** sp., Nangongshan Forest Park, Shaanxi, China**	**KF769413**	**KF769400**	**KF769424**
	AS3.15372	Plant leaves, Gongbujiangda, Linzhi, Tibet, China	KF769417	KF769404	KF769428
*P. glabrum* Thom	CBS 105.11	Ex-type of *P. frequentans*; unknown substrate, Germany	GQ367525	GQ367501	GU981567 (CBS 125543^T^)
	CBS 229.28	Ex type of *P. paczowskii*; soil under conifer, Poland	GQ367531	GQ367506	N/A
	NRRL 35621	Cork bark, southern Portugal	EF198575	EF198547	N/A
	NRRL 35684	Cork bark, southern Portugal	EF198592	EF198564	N/A
	AS3.15335	Leaves of *Rhododendron* sp., Cona County, Tibet, China	KF302640	KF302630	KF302650
	AS3.15336	Plant leaves, Tiantaishan Forest Park, Shaanxi, China	KF769418	KF769405	KF769429
	AS3.15337	Leaves of *Rhododendron* sp., Nangongshan Forest Park, Shaanxi, China	KF769419	KF769406	KF769430
	AS3.15345	Leaves of *Quercus palustris*, Nangongshan Forest Park, Shaanxi, China	KF302645	KF302635	KF302655
*P. grancanariae* C. Ramírez, A.T. Martínez & Ferrer	CBS 687.77^T^	Air, Gran Canaria, Spain	GQ367533	GQ367507	N/A
	CBS 336.79	Ex-type of *P. palmense*, air, Gran Canaria, Spain	GQ367534	GQ367508	N/A
*P. herquei* Bainier & Satory	CBS 336.48^T^ = NRRL 1040^T^	A leaf of *Agauria pyrifolia*, France	JN626013	JN625970	AF033405
*P. hirayamae* Udagawa	NRRL 143 ^T^ = CBS 229.60^T^	Milled Thai rice, Japan	EU021691	JN625955	JN626095
*P. lividum* Westling	IMI 39736 ^T^ = NRRL 754^T^	Unrecorded source, Scotland	DQ911124	FJ004420	AF033406
	AS3.15334	Cona County, Tibet, China	KF769420	KF769407	KF769431
*P. maximae* C.M. Visagie, J. Houbraken & R.A. Samson	NRRL 2060^T^	Cellulose nitrate coated cellophane, Florida, USA	EU427282	EU427265	EU427298
*P. purpurascens* (Sopp) Biourge	IMI 39745^T^ = CBS 366.48^T^ = NRRL 720^T^	Soil, Canada	DQ911125	GQ367512	AF033408
*P. saturniforme* (L. Wang & W.Y. Zhuang) Houbraken & Samson	AS3.6886^T^	Soil, Little Peony Forest Reserve, Dunhua, Jilin Province, China	EU644062	EU644080	EU644081
*P. sclerotiorum* van Beyma	NRRL 2074^T^	Air, Buitenzorg, Java, Indonesia	JN626044	JN626001	JN626132
*P. spinulosum* Thom	IMI 24316i^T^ = AS3.7980^T^	Hanover, Germany	DQ911126	KF769408	KF769432
	CBS 223.28	N/A	GQ367536	GQ367509	N/A
	CBS 268.35	Ex-type of *P. mediocre*; soil, pine forest; Germany	GQ367527	GQ367499	N/A
	CBS 271.35	Ex-type of *P. tannophilum*; leaf litter,Germany	GQ367530	GQ367505	N/A
	CBS 289.36	Ex-type of *P. tannophagum*; tannin solution,Germany	GQ367528	GQ367503	N/A
*P. subericola* Barreto, Frisvad & Samson	CBS 125096^T^	N/A	GQ367547	GQ367521	N/A
	CBS 125100	Dried grapes (sultanas, *Vitis vinifera*), Mildura, Vic, Australia	GQ369760	GQ369759	N/A
*P. thomii* Maire	IMI 189694^T^ = AS3.7982^T^	Pine cone, unknown country	DQ911127	KF769409	KF769433
“*P. thomii* var. *flavescens*” S. Abe	CBS 347.59^T^	from unrecorded substrate, Japan	GQ367535	GQ367510	N/A
	AS3.15339	Plant leaves, Tiantaishan Forest Park, Shaanxi, China	KF769414	KF769401	KF769425
	AS3.15346	Leaves of *Betula utilis*, Heihe Forest Park, Shaanxi, China	KF769415	KF769402	KF769426
	AS3.15371	Plant leaves, Gongbujiangda, Linzhi, Tibet, China	KF769416	KF769403	KF769427
“*P. yezoense*” Hanzawa in Sasaki & Nakane	CBS 350.59^T^	Butter, Japan	GQ367548	GQ367517	N/A
*P. species* (related to “*P. yezoense*”)	AS3.15349	Plant leaves, Tiantaishan Forest Park, Shaanxi, China	KF769421	KF769410	KF769434
***P. zhuangii*** ** L. Wang sp. nov.**	**AS3.15341^T^**	**Leaves of ** ***Betula utilis*** **, Heihe Forest Park, Shaanxi, China**	**KF769422**	**KF769411**	**KF769435**
	AS3.15347	Leaves of *Rhododendron* sp., Nangongshan Forest Park, Shaanxi, China	KF769423	KF769412	KF769436

*AS, China General Microbiological Culture Collection, Academia Sinica, Beijing, China; CBS, Centraalbureau voor Schimmelcultures, Utrecht, The Netherlands; IMI, International Mycological Institute, Surrey, UK; NRRL, Agricultural Research Service Culture Collection, Illinois, USA; ex-type strains are indicated with ^T^.

# Sequences KF769400-KF769436 obtained in present study.

### Nomenclature

The electronic version of this article in Portable Document Format (PDF) in a work with an ISSN or ISBN will represent a published work according to the International Code of Nomenclature for algae, fungi, and plants, and hence the new names contained in the electronic publication of a PLOS ONE article are effectively published under that Code from the electronic edition alone, so there is no longer any need to provide printed copies.

In addition, new names contained in this work have been submitted to MycoBank from where they will be made available to the Global Names Index. The unique MycoBank number can be resolved and the associated information viewed through any standard web browser by appending the MycoBank number contained in this publication to the prefix http://www.mycobank.org/MB/. The online version of this work is archived and available from the following digital repositories: PubMed Central and LOCKSS.

## Results

### Phylogenetic delineation of *P. fusisporum* and *P. zhuangii*

PCR amplification gave amplicons of *CaM* about 680 bp, *BenA* about 680 bp using primers I2 and Bt2b, and ca. 450 bp using primers Bt2a and Bt2b, and *ITS1-5.8S-ITS2* about 560 bp. The trimmed alignments of *CaM*, *BenA* and *ITS1-5.8S-ITS2* sequences were respectively 507, 475 and 536 characters with gaps.

**Figure 1:**
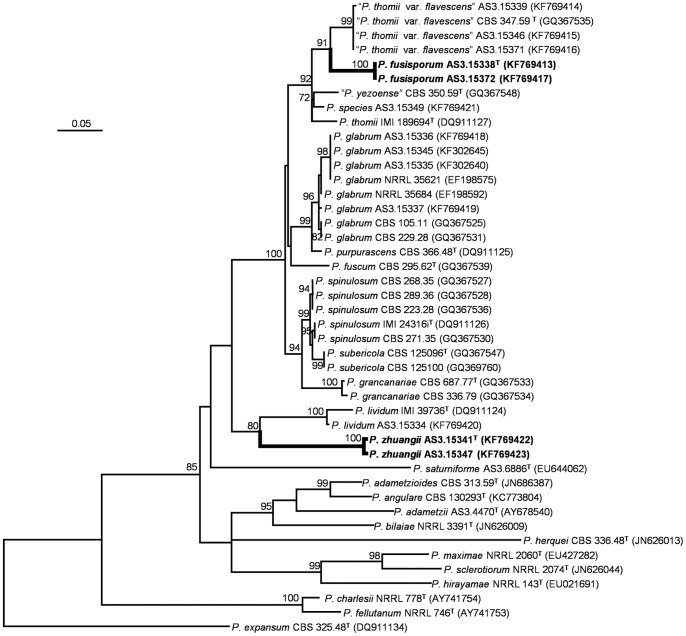
ML phylogram inferred from partial *CaM* sequences. Bootstrap percentages over 70% derived from 1000 replicates are indicated at the nodes. Bar  = 0.05 substitutions per nucleotide position.

**Figure 2:**
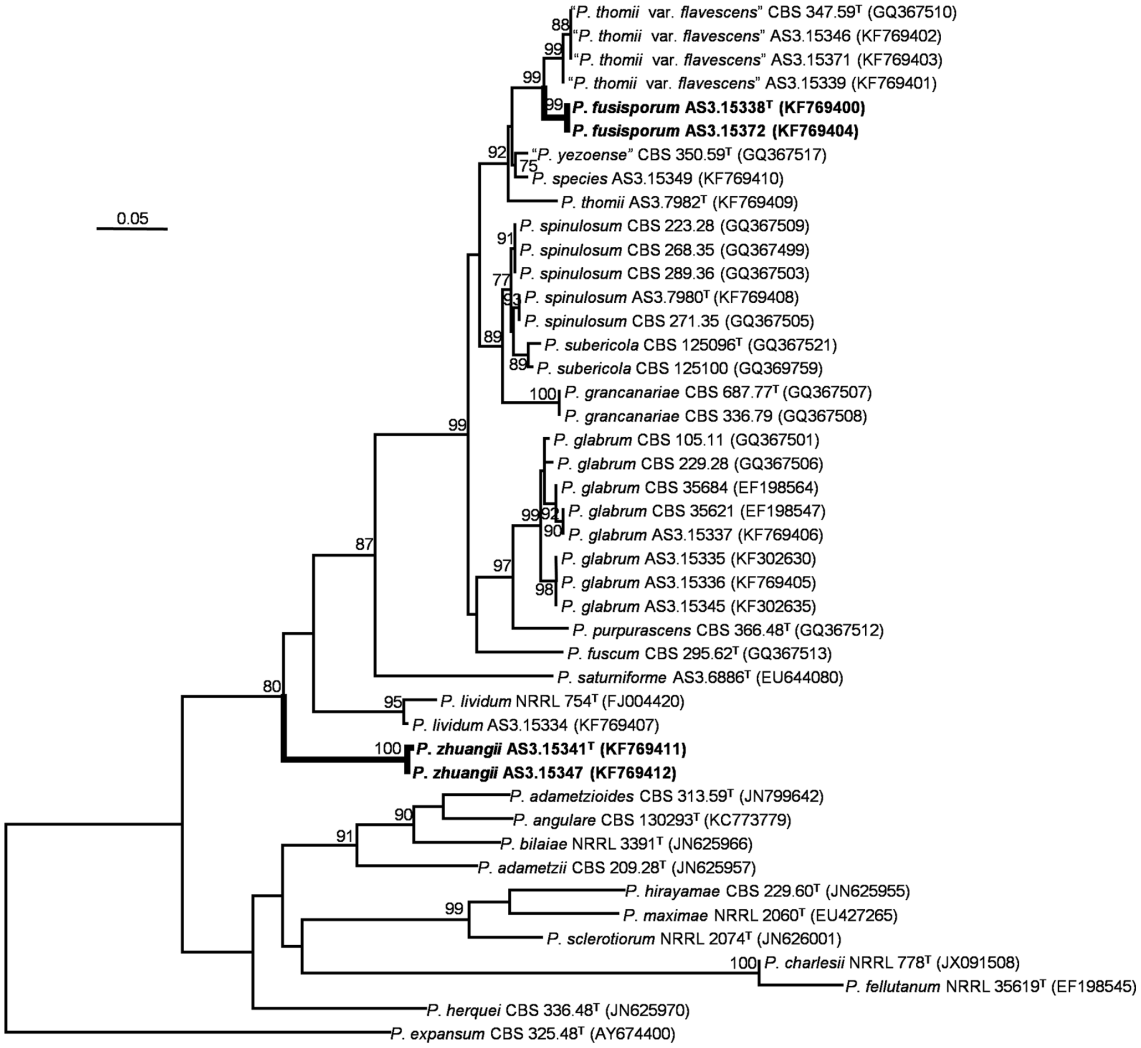
ML phylogram inferred from partial *BenA* sequences. Bootstrap percentages over 70% derived from 1000 replicates are indicated at the nodes. Bar  = 0.05 substitutions per nucleotide position.

The phylograms resulting from *CaM* and *BenA* matrices showed that *P. fusisporum* was closely related to “*P. thomii* var. *flavescens*” with 91% and 99% bootstrap support, respectively. While *P. zhuangii* was in a clade related to *P. lividum* with 80% bootstrap support based on the *CaM* analysis, but in the phylograms based on the *BenA* and *ITS1-5.8S-ITS2* regions, *P. zhuangii* formed a separate clade without close relatives. The phylogenetic trees generated by the three loci supported them as distinct, monophyletic species (Figures 1–2; Figure S1).

### Description of *Penicillium fusisporum* L. Wang, sp. nov. Figures 3–4

**Figure 3:**
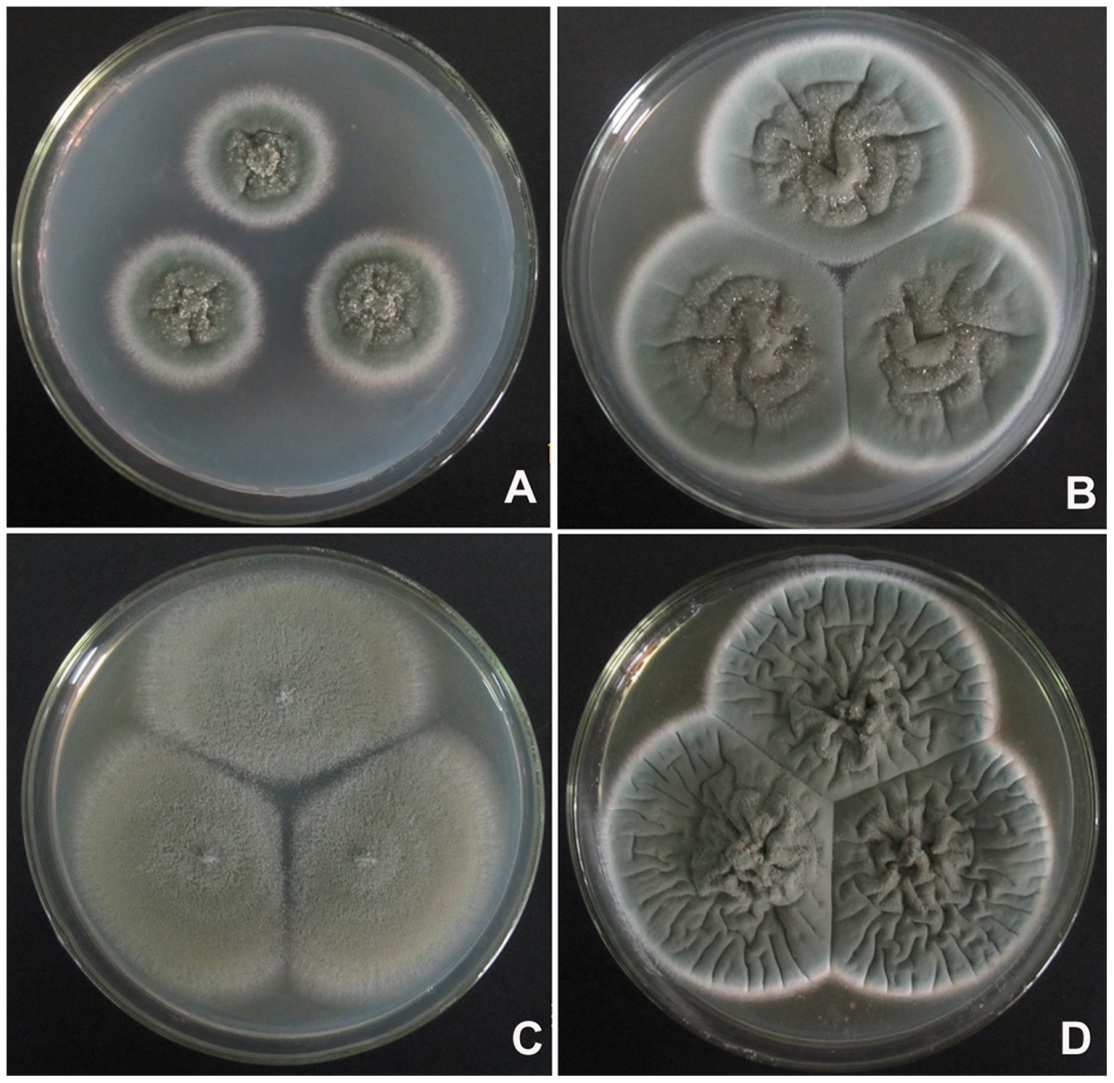
Colonies of *P. fusisporum* AS3.15338 ^T^ incubated 7 d at 25°C. A, Cz; B, CYA; C, MEA; D, YES.

**Figure 4:**
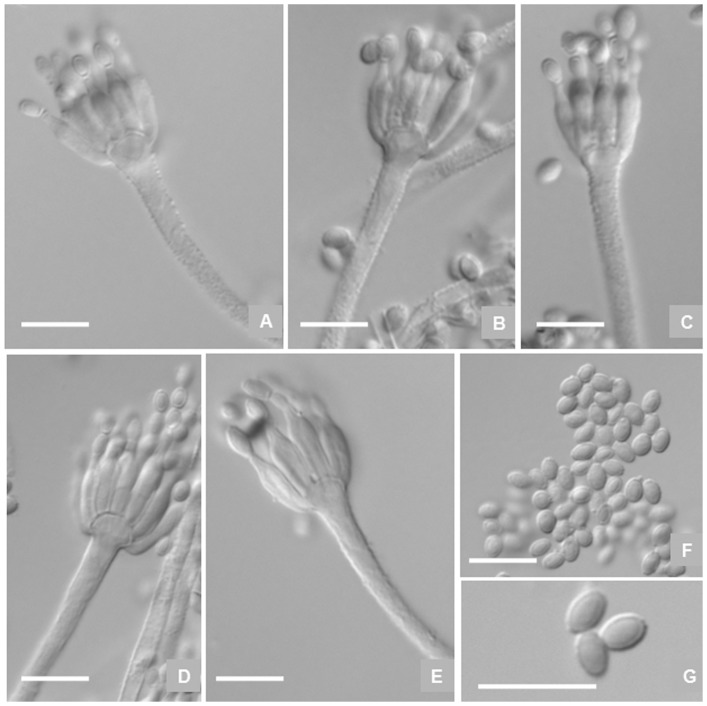
Microscopic characters of *P. fusisporum* AS3.15338 ^T^. A–E, Conidiophores; F–G, Conidia. Bar  = 10 µm.

[urn:lsid:indexfungorum.org:names: 806119], MycoBank MB 806119

Etymology: The specific epithet is derived from the fusiform shape of its conidia.

Holotype: HMAS 244961

Colonies 33–34 mm diam on Cz at 25°C after 7 d, thin, radially sulcate in central areas, umbonate in centers; sclerotia moderate in centers, white when young, then Cinnamon (R. Pl. XXIX) when mature; velutinous; conidiogenesis moderate to abundant, near Pea Green to Sage Green (R. Pl. XLVII); mycelia white; clear exudate limited; soluble pigment absent; reverse Cinnamon to Verona Brown or Warm Sepia in centers, but showing Pale Pinkish Buff at periphery (R. Pl. XXIX). Colonies 50–53 mm diam on CYA at 25°C after 7 d, thin, umbonate or protuberant in centers, irregularly sulcate; sclerotia moderate in centers, white when immature, then Cinnamon (R. Pl. XXIX); velutinous; conidiogenesis abundant, near Pea Green to Sage Green (R. Pl. XLVII); mycelium white; clear exudate moderate in central areas and no soluble pigment; reverse Verona Brown in centers but Pinkish Buff at periphery (R. Pl. XXIX). Colonies 58–60 mm diam on MEA at 25°C after 7 d, low, plane, slightly protuberant in centers; velutinous; conidiogenesis abundant, near Sage Green (R. Pl. XLVII); no exudate and soluble pigment; reverse Deep Olive-Buff (R. Pl. XL). Colonies 55–57 mm diam on YES at 25°C after 7 d, thin, protuberant or convolute in centers, irregularly sulcate; Cinnamon (R. Pl. XXIX) sclerotia abundant in centers; velutinous; conidiogenesis abundant, Grayish Olive (R. Pl. XLVI); exudate and soluble pigment absent; reverse Colonial Buff (R. Pl. XXX). On G25N at 25°C after 7 d, colonies 7–8 mm diam, thin, radially sulcate moderate; velutinous; with moderate conidiogenesis colored Light Grayish Olive (R. Pl. XLVI); no exudate and soluble pigment; reverse Plae Ochraceous-Salmon ((R. Pl. XV). On CYA at 37°C after 7 d, no growth. On CYA at 5°C after 7 d, germination only.

Sclerotia commonly produced on Cz, CYA and YES, first colored white then Cinnamon when mature, irregular in shape, up to 600 µm in the long axis. Conidiophores arising from agar surface; stipes (90–) 120–200 (−250)×2.5–3.5 µm, verrucose, seldom smooth-walled, apically swelling up to 4–6 µm diam; penicilli monoverticillate, but occasionally with one branch about 12–20 µm long on CYA; phialides (8–) 12–16 per verticil, ampulliform with distinguishable collula, 9–11×2–3 µm; conidia fusiform to oblong, 3.5–4×2–2.5 µm, walls smooth, conidial chains irregularly tangled about 120–180 µm long.

#### Strains examined

China, Shaanxi, Nangongshan Forest Park, 31°45′35″N 110°38′57″E, 1500 m, from leaves of *Rhododendron* sp., 6 Oct 2012, coll. P-J Han, leaf samples no. NGS13E, ex-type culture AS3.15338 (Holotype: HMAS 244961 from dried culture of ex-type AS3.15338 on CYA). Tibet, Linzhi, Gongbujiangda, 29°54′43″N 93°10′43″E, 3400 m, from unidentified plant leaves, 12 Aug 2012, coll. P-J Han, leaf samples no. GBJD3D, additional culture AS3.15372.

#### Notes

This new species is characterized by its fast growth rate, cinnamon-colored sclerotia, apical-swelling monoverticillate conidiophores,verrucose stipes, and fusiform to oblong smooth-walled conidia.

### Description of *Penicillium zhuangii* L. Wang, sp. nov. Figures 5–6

**Figure 5:**
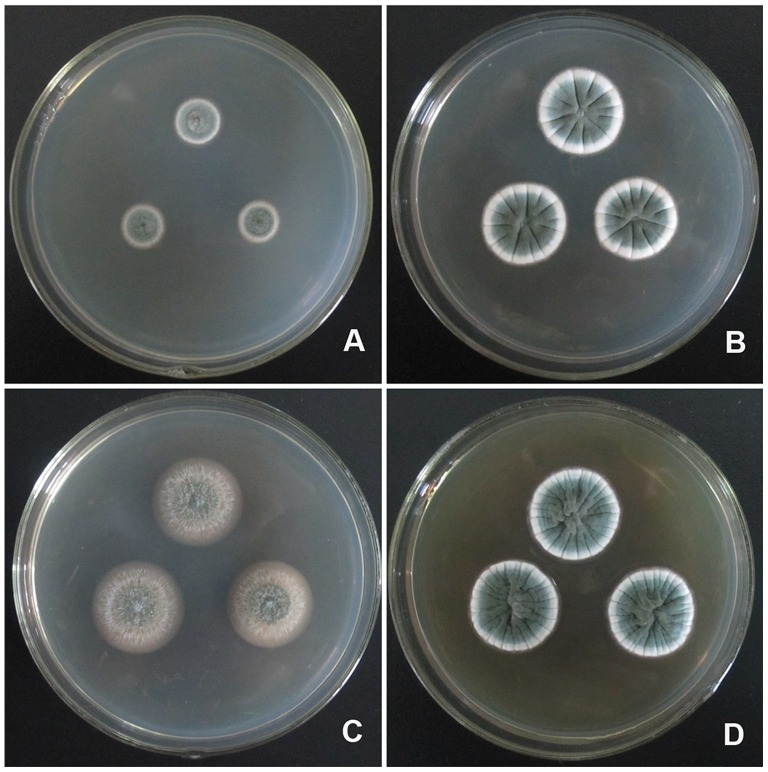
Colonies of *P. zhuangii* AS3.15341 ^T^ incubated 7 d at 25°C. A, Cz; B, CYA; C, MEA; D, YES.

**Figure 6:**
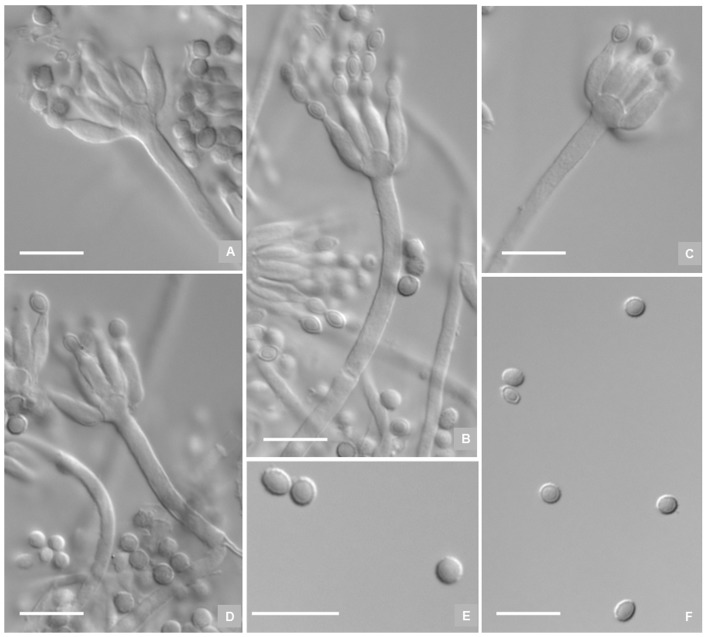
Microscopic characters of *P. zhuangii* AS3.15341 ^T^. A–D, Conidiophores; E–F, Conidia. Bar  = 10 µm.

[urn:lsid:indexfungorum.org:names: 805945], MycoBank MB 805945

Etymology: named after Prof. Jian-Yun Zhuang, who made great contribution to the taxonomy of Pucciniales in China.

Holotype: HMAS 244922

Colonies 12–14 mm diam on Cz at 25°C after 7 d, thin, plane, protuberant in central areas; velutinous; conidiogenesis moderate, near Pea Green to Celandine Green (R. Pl. XLVII); mycelium white; no exudate and soluble pigment; reverse Light Cinnamon-Drab to Cinnamon Drab (R. Pl. XLVI). Colonies 23–27 mm diam on CYA at 25°C after 7 d, thin, radially sulcate; velutinous; conidiogenesis moderate, near Pea Green to Andover Green (R. Pl. XLVII); mycelium white; no exudate or soluble pigment; reverse Pale Ochraceous-Buff (R. Pl. XV). Colonies 24–26 mm diam on MEA at 25°C after 7 d, low, plane, margins submerged in agar; velutinous, with sparsely floccose white mycelia overlaid; conidiogenesis limited to moderate, near Grayish Olive (R. Pl. XLVI); no exudate or soluble pigment; reverse Tawny-Olive (R. Pl. XXIX). Colonies 24–26 mm diam on YES at 25°C after 7 d, thin, convolute in centers, radially sulcate and lightly annually plicate; velutinous; conidiogenesis abundant, Andover Green or Pea Green to Celandine Green (R. Pl. XLVII); exudate absent; slightly Ochraceous-Tawny (R. Pl. XV) soluble pigment limited; reverse Tawny to Ochraceous-Tawny (R. Pl. XV). On G25N at 25°C after 7 d, colonies 2–5 mm diam, with limited conidiogenesis colored Light Grayish Olive (R. Pl. XLVI). On CYA at 37°C after 7 d, no growth. On CYA at 5°C after 7 d, colonies 2–6 mm diam, with white mycelium only.

Conidiophores arising from agar surface; stipes (120–) 140–180 (−220)×2.5–3.6 µm, smooth-walled, apically swelling up to 3.5–5.5 µm diam; penicilli exclusively monoverticillate; phialides 8–12 per verticil, ampulliform with distinguishable collula, (7–) 9–13×2.5–3.5 µm; conidia ovoid to globose, 3–3.5 µm, walls smooth, born in irregularly tangled chains about 100–140 µm long.

#### Strains examined

China, Shaanxi, Zhouzhi Heihe Forest Park, 33°52′54″N 107°47′51″E, 1200 m, from leaves of *Betula utilis*, 28 Sep 2012, coll. P-J Han, leaf samples no. ZHH20, ex-type culture AS3.15341 (Holotype: HMAS 244922, from dried culture of ex-type AS3.15341 on CYA). Shaanxi: Nangongshan Forest Park, 31°45′35″N 110°38′57″E, 1500 m, from leaves of *Rhododendron* sp., 6 Oct 2012, coll. P-J Han, leaf samples no. NGS13D, additional culture AS3.15347.

#### Notes

This new taxon is characterized by its moderate growth rate, apical-swelling monoverticillate conidiophores, smooth-walled stipes, and ovoid to subglobose smooth-walled conidia.

## Discussion

The two new taxa reported here should be placed in *Penicillium sensu stricto* section *Aspergilloides* in the classification of the most recent phylogenetic scheme by Houbraken and Samson [6].

Raper and Thom regarded the strains with monoverticillate penicilli and hard but brittle sclerotia in pink color as *P. thomii*. Abe [18] reported a new variety of *P. thomii*, namely “*P. thomii* var. *flavescens*” (*nom. inval*., Art. 36), which differs from *P. thomii* by its fast growth rate, abundant Pinkish Cinnamon sclerotia and the yellow-green colony color. Pitt [2] and Pitt *et al.* [3] broadened the concept of *P. thomii* to include *P. aurantioviolaceum*, *P. crocicola*, *P. roseoviride*, “*P. thomii* var. *flavescens*”, and “*P. yezoense*” (*nom. inval*., Art. 36). However in our phylogenies based on *CaM* and *BenA*, the type culture CBS 347.59 of “*P. thomii* var. *flavescens*” together with our three strains forms a monophyletic lineage well separated from the type culture IMI 189694 of *P. thomii*, which means that “*P. thomii* var. *flavescens*” represents a different species other than *P. thomii* (Figures 1–2). In addition, “*P. thomii* var. *flavescens*” is different from *P. thomii* in many morphological aspects. For instance, the isolates of “*P. thomii* var. *flavescens*” produce both smooth-walled and sparsely granular conidiophores, while those of *P. thomii* are finely roughened. Moreover, the conidia of “*P. thomii* var. *flavescens*” also show subglobose to ovoid shape in addition to ellipsoidal shape, but those of *P. thomii* are ellipsoidal.

Although isolates of *P. fusisporum* and “*P. thomii* var. *flavescens*” grow rapidly on standard media and produce sclerotia colored near Cinnamon, isolates of “*P. thomii* var. *flavescens*” excretes pale yellow-green soluble pigment on Cz and its colony reverse also shows the same tint, but *P. fusisporum* does not. Besides, the conidial of *P. fusisporum* colored near Pea Green to Sage Green, instead of Bottle Green or Russian Green in”*P. thomii* var. *flavescens*”. Still, the stipe walls of *P. fusisporum* are commonly verrucose and conidia are fusiform to oblong, whereas, stipe walls of isolates in “*P. thomii* var. *flavescens*” are delicately or sparsely granular to smooth and its conidial shape is subglobose or ovoid to ellipsoidal. Our phylogenies based on *CaM* and *BenA* both show the kinship between them (91% and 99% bootstrap support respectively), but also indicate that *P. fusisporum* forms a distinct clade basal to those isolates “*P. thomii* var. *flavescens*” (Figures 1–2).

Raper and Thom [1] distinguished *P. aurantioviolaceum* Biourge from *P. thomii* for its nonsclerotigenic characters and narrow- ellipsoidal to fusiform conidia, and treated *P. roseoviride* Stapp and Bortels as the synonym of it. Although Pitt [2] and Pitt *et al.* [3] did not follow this practice, from the characters described by Raper and Thom [1], we think *P. aurantioviolaceum* would be regarded as a separate species. Though both *P. fusisporum* and *P. aurantioviolaceum* have monoverticillate penicilli with occasionally branched stipes and narrowly ellipsoidal to fusiform conidia, *P. fusisporum* shows a velutinous colony texture on all standard media and bears abundant sclerotia, while *P. aurantioviolaceum* gives a loosely velutinous to floccose texture and never produces sclerotia. Moreover, the stipe length of *P. fusisporum* is much shorter (less than 250 µm), nearly one half of those of *P. aurantioviolaceum*, which are at least 400 µm, and the stipe walls of *P. fusisporum* are commonly verrucose, but those of *P. aurantioviolaceum* are closely and finely echinulate. Furthermore, *P. fusisporum* produces smooth-walled conidia, whereas, those of *P. aurantioviolaceum* are predominantly delicately roughened.

Also, Pitt [2] and Pitt et al. [3] relegated *P. crocicola* as a synonym of *P. thomii*, but Houbraken and Samson [6] confirmed its species status, and showing its outgroup position to *P. patens* and “*P. thomii*” CBS 347.59. While *P. fusisporum* is unlikely conspecific with *P. crocicola*, because in our phylogram of ITS1-5.8S-ITS2 (Figure S1) P. fusisporum is much more closely related to “*P. thomii* var. *flavescens*” than *P. crocicola* is, and above all, P. crocicola is the outgroup of *P. aurantioviolaceum*, “*P. thomii* var. *flavescens*”, *P. fusisporum*, *P. purpurascens*, *P. thomii*, and *P. trzebinskii* Zaleski as well as *P. glabrum*. Given the fact that ITS1-5.8S-ITS2 and the DNA-dependent RNA polymerase II second largest subunit gene (RPB2) are more evolutionarily conserved than CaM and BenA in penicillia, it could be inferred that *P. fusisporum* would be much distant to *P. crocicola*. In addition to the molecular evidence, there are clear differences between *P. fusisporum* and *P. crocicola* referring to the translated diagnosis of by Kulik [20]. For example, *P. fusisporum* produces longer stipes up to 250 µm than those of *P. crocicola* which are about 106 µm at most. Moreover, the stipe walls of *P. fusisporum* are verrucose but those of *P. crocicola* are smooth. The penicilli of *P. crocicola* are monoverticillate, while *P. fusisporum* bears sub-terminal branches on CYA. Furthermore, the conidia of *P. fusisporum* are fusiform to oblong, whereas, those of *P. crocicola* are subglobose to globose. Still more, the sclerotia of *P. fusisporum* are about twice longer (up to 600 µm) as those of P. crocicola which are about 320 µm in the long axis.

The Pea Green conidial color, abundant sclerotia and vesiculate monoverticillate penicilli of *P. fusisporum* also resemble those of *P. thomii*, however, the difference between them is obvious. Firstly, *P. fusisporum* grows faster and produces larger sclerotia up to 600 µm long and in a darker color near Cinnamon, while the growth rate of *P. thomii* is slower, the sclerotia are less than 350 µm long and in Apricot or Salmon shade. Secondly, *P. fusisporum* occasionally bears one sub-terminal branch on CYA, but those of *P. thomii* are strictly monoverticillate. Thirdly, the stipe walls of P. fusisporum are apparently verrucose whereas, those of *P. thomii* are delicately roughened. Still, the new taxon produces fusiform to oblong conidia, nonetheless, *P. thomii* bears ellipsoidal conidia.

Pit and Hocking [19] reported a new species producing sclerotia and long, roughened stipes bearing monoverticillate penicilli, *P. patens* Pitt and Hocking. In the phylogram of Houbraken and Samson [6], *P. patens* was in the clade with”*P. thomii*” CBS 347.59 with a significant bootstrap support, just like the relationship of *P. fusisporum* with the four strains of “*P. thomii* var. *flavescens*” including CBS 347.59 in our phylograms. Although *P. fusisporum* and *P. patens* are alike in colony appearance, *P. fusisporum* can be readily distinguished from *P. patens* in many aspects. First, the sclerotia of *P. fusisporum* are much larger (up to ca. 600 µm of the long axis) than those of *P. patens* (up to 300 µm in the long axis). Second, *P. fusisporum* produces much shorter stipes less than 250 µm long, than those of *P. patens*, which are up to 500–800 µm. Third, the stipe walls of *P. fusisporum* are verrucose, while those of *P. patens* are finely to conspicuously roughened, and the stipes are apical-swelling in *P. fusisporum* but *P. patens* bears nonvesiculate stipes. Still, though *P. fusisporum* produces predominantly monoverticillate penicilli, it occasionally bears sub-terminal branches on CYA, whereas, *P. patens* produces strictly monoverticillate penicilli. Furthermore, the conidial shapes of *P. fusisporum* are fusiform to oblong, but those of *P. patens* are ellipsoidal [19]. All the evidence discussed above supported the new species status of *P. fusisporum*. The comparisons of the key characters of the above species are summarized in Table 2.

**Table 2:** Comparisons of microscopic characters among *P. aurantioviolaceum*, *P. crocicola*, *P. fusisporum*, *P. patens*, *P. thomii* and “*P. thomii* var. flavescens” *.

	*P. aurantioviolaceum*	*P. crocicola*	*P. fusisporum*	*P. patens*	*P. thomii*	“*P. thomii* var. *flavescens*”
Sclerotia (µm)	None	Yellow-brown to dark brown, 140–320	**Cinnamon, up to 600**	Colorless to pale brown, 130–200×130–150	Pinkish to apricot color, 250–350	Pinkish cinnamon to salmon orange, 190–510×150–410
Conidiophores						
Stipes (µm)	Closely and finely echinulate, 400 or more ×2.5–3.5,	Smooth, 32–106×3–4	**Verrucose, seldom smooth-walled, (90–) 120–200 (−250)×2.5–3.5**	Finely to conspicuously roughened, 500–800×2.5–3	Delicately echinulate, 200–400×2.8–4	Delicately or sparsely granular, 90–300×2.1–3.4
Apical swellings or vesicles (diam., µm)	4.5–5	N/A	**4–6**	None	4–6	3.7×6.2
Penicilli	Strictly monoverticillate	Monoverticillate	**Monoverticillate, occasionally with one subterminal branch on CYA**	Strictly monoverticillate	Monoverticillate, seldom branched	Strictly monoverticillate
Phialides (µm)	8–10×2.0–2.5	7–14×2.5–3.5	**9–11×2–3**	8–10×2.5–3	8–12×2–3	9.3–10.6×1.7–2.5
Conidia (µm)	Strongly ellipsoidal to fusiform, 3–3.5×2–2.5	Subglobose to globosel, 2.3–3.5×2–3	**Fusiform to oblong, 3.5–4×2–2.5**	Ellipsoidal, 3–3.5×2–2.5	Ellipsoidal to subglobose, 3–3.5(−4)×2.5–2.8	Ellipsoidal to subglobose, 2.8–3.6×1.7–2.1
Conidial walls	Delicately roughened	Smooth	**Smooth**	Smooth	Smooth or finely to conspicuously oughend	Smooth

*These data were integrated from our observations, Raper and Thom (1949), Abe (1956), Kulik (1968), Pitt (1979), Pitt and Hocking (1985).

The designation of *P. zhuangii* as a new species is supported by our all phylograms inferred from *CaM*, *BenA* and *ITS1-5.8S-ITS2* loci (Figures 1–2; Figure S1). The phylogenies inferred from the three loci indicate that *P. zhuangii* is a well-separated species with no closely related kins. Although *CaM* sequence datum slightly show that *P. zhuangii* might be a sibling of *P. lividum* (with only 80% bootstrap support), it can be discriminated from *P. lividum* by many morphological characters such as growth rate, conidiophore elements and conidia. The growth rate of *P. zhuangii* is characteristically slower than those of typical isolates of *P. lividum* (commonly faster than 30 mm on standard media) and stipe length of *P. zhuangii* is much shorter, less than 250 µm, than most of *P. lividum* isolates, which are 400–600 µm. In addition, the stipe walls of the *P. zhuangii* are exclusively smooth, while some strains of *P. lividum* show distinctively rough walls. Moreover, *P. zhuangii* bears only monoverticillate conidiophores, whereas certain strains of *P. lividum* produce short branches reminiscent of metulae. Furthermore, the conidia of *P. zhuangii* are smooth-walled, while, those of *P. lividum* are characteristically roughened.

Pitt [2] and Pitt et al. [3] listed P. odoratum Christensen and Backus and “*P. trzebinskianum*” as the synonyms of *P. lividum*, but in the phylogenetic tree of Houbraken and Samson [6], the sister species to *P. lividum* is *P. odoratum*. And our phylogeny based on *ITS1-5.8S-ITS2* does not show any close relationship between *P. zhuangii* and *P. odoratum* and “*P. trzebinskianum*” (Fig. S1). In addition, *P. zhuangii* can be distinguished from *P. odoratum* due to many distinctive characters. First, *P. zhuangii* shows moderate conidiogenesis and does not emit fruity odor, while *P. odoratum* generates conidia tardily and giving off strong aromatic odor like apples, though this was regarded as an ephemeral character by Pitt [2]. Second, the conidiophores of *P. zhuangii* are much shorter (less than 220 µm) than those of *P. odoratum* which are about 560 µm long, and the stipe walls of *P. zhuangii* are smooth but those of *P. odoratum* are roughened. Third, the apical swellings of stipes in *P. zhuangii* are much smaller (about 5.5 µm) than those of *P. odoratum* (up to 7–9 µm in diam.). Still, though both species bear ovoid to globose conidia, the conidial walls of *P. zhuangii* are smooth, yet those of *P. odoratum* are roughened and showing faintly banded [21]. In addition, *P. zhuangii* can also be readily distinguished from “*P. trzebinskianum*” morphologically in that *P. zhuangii* grows slower and its conidial color is near Pea Green, however “*P. trzebinskianum*” grows much faster and its conidial color is near Dusky Dull Green. Moreover, *P. zhuangii* produces smooth-walled conidiopores but those of “*P. trzebinskianum*” are punctate or granular. Further, the conidia of *P. zhuangii* are smooth-walled whereas, those of “*P. trzebinskianum*” are echinulate [20].

Thom and Raper [1] also treated *P. trzebinskii*, a slowly-growing species with short, monoverticillate conidiophores as a valid taxon other than *P. lividum*. However, Pitt (1979), relegated it to one synonym of *P. spinulosum*. The phylogram inferred from *ITS1-5.8S-ITS2* in our studies does not only show that *P. trzebinskii* and *P. spinulosum* are different species, but also shows that *P. zhuangii* is far distant to *P. trzebinskii* (Fig. S1). In addition to this phylogenetic evidence, *P. zhuangii* can be readily differentiated from *P. trzebinskii* in that the walls of stipes and conidia are both smooth in *P. zhuangii*, while those of *P. trzebinskii* are conspicuously echinulate. Besides, the colony appearance on Cz of *P. zhuangii* shows a velutinous texture without any overlaid mycelia appearing floccose, but *P. trzebinskii* does, and the reverse color of colonies in *P. zhuangii* shows Cinnamon Drab, but that of *P. trzebinskii* gives deep dull violet to dark fuscous color.

Pitt et al. [3] regarded *P. quercetorum* Baghdadi as the synonym of *P. thomii*, but in the phylogram of Houbraken and Samson [6], *P. quercetorum* is an outgroup species to *P. lividum* and *P. odoratum*, and our *ITS1-5.8S-ITS2* phylogram does not only indicates that *P. quercetorum* is much distant to *P. thomii* but also shows its outgroup position to *P. zhuangii* (Fig. S1). Though the growth rate and certain microscopic characters of *P. quercetorum* and *P. zhuangii* are overlapped, the most striking difference between them is that *P. quercetorum* produces orange-brown sclerotia, while no sclerotia were produced by *P. zhuangii*. Additionally, *P. quercetorum* produces much longer stipes (200–400 µm) than *P. zhuangii* (less than 220 µm), and it also produces smaller conidia (less than 3.0 µm) than *P. zhuangii* (3–3.5 µm). According to the above evidence, *P. zhuangii* should be regarded as a valid new taxon. The comparisons of the key characters of the above species are summarized in Table 3.

**Table 3:** Comparisons of microscopic characters among *P. lividum*, *P. odoratum*, *P. quercetorum*, “*P. trzebinskianum*”, *P. trzebinskii* and *P. zhuangii* *.

	*P. lividum*	*P. odoratum*	*P. quercetorum*	“*P. trzebinskianum”*	*P. trzebinskii*	*P. zhuangii*
Sclerotia (µm)	None	None	Orange brown, 200–300	None	None	**None**
Conidiophores						
Stipes (µm)	Smooth, 400–600 or more ×2.5–4	Coarsely roughened, 100–560×3–4	Smooth, 200–400×3–3.5	Punctate or granullar, 60–280×2.5–4	Delicately and conspicuously echinulate, 150–200×1.8–2.5	**Smooth, (120–) 140–180 (−220)×2.5–3.6**
Apical swellings or vesicles (diam., µm)	5–6	7–9	6	3.7–6.9	N/A	**3.5–5.5**
Penicilli	Monoverticillate, occasionally with a branch	Monoverticillate	Strictly monoverticillate	Strictly monoverticillate	Monoverticillate	**Exclusively** **monoverticillate**
Phialides (µm)	8–12×2–3	9–12×3–4	8–12×2.8–3	8.7–12.5×2.1–3.2	8–10×1.8–2.2	**(7–) 9–13×2.5–3.5**
Conidia (µm)	Ellipsoidal to ovoid or subglobose, 3–4×2.6–3	Ellipsoidal to ovoid or subglobose, 3–4.1×2.2–4	Spheroidal, 2.8–3	Ellipsoidal to ovoid or sometimes subglobose, 2.5–3.8×2.3–3.1	Broadly ellipsoidal to subglobose, 2.5–3.3	**Ovoid to globose, 3–3.5**
Conidial walls	Clearly roughened showing spiral banding	Roughened appearing faintly banded	Smooth	Echinulate	Conspicuously echinulate	**Smooth**

*These data were integrated from our observations, Raper and Thom (1949), Christensen and Backus (1956), Kulik (1968), Pitt (1979).

## Supporting Information

Figure S1 **ML phylogram inferred from the** ***ITS1-5.8S-ITS2*** **sequences.** Bootstrap percentages over 70% derived from 1000 replicates are indicated at the nodes. Bar  = 0.02 substitutions per nucleotide position. The sequences of *P. odoratum* NBRC 7741 ^T^, “*P. trzebinskianum*” NBRC 6038 ^T^ and *P. trzebinskii* NBRC 6110 ^T^ were retrieved from the on-line catalogue of Biological Resource Center (NBRC), NITE of Japan (http://www.nbrc.nite.go.jp/e/).
(TIF)

Data S1 The alignment of *CaM* sequences.
(FAS)

Data S2 The alignment *of BenA* sequences.
(FAS)

Data S3 The alignment *of ITS1-5.8S-ITS2* sequences.
(FAS)
