# Supplementary figures and images for: Penicillium fusisporum and P. zhuangii, Two New Monoverticillate Species with Apical-Swelling Stipes of Section Aspergilloides Isolated from Plant Leaves in China
Source: PLoS One. 2014 Jul 2;9(7):e101454. doi: 10.1371/journal.pone.0101454 (PMC4079715; doi:10.1371/journal.pone.0101454)

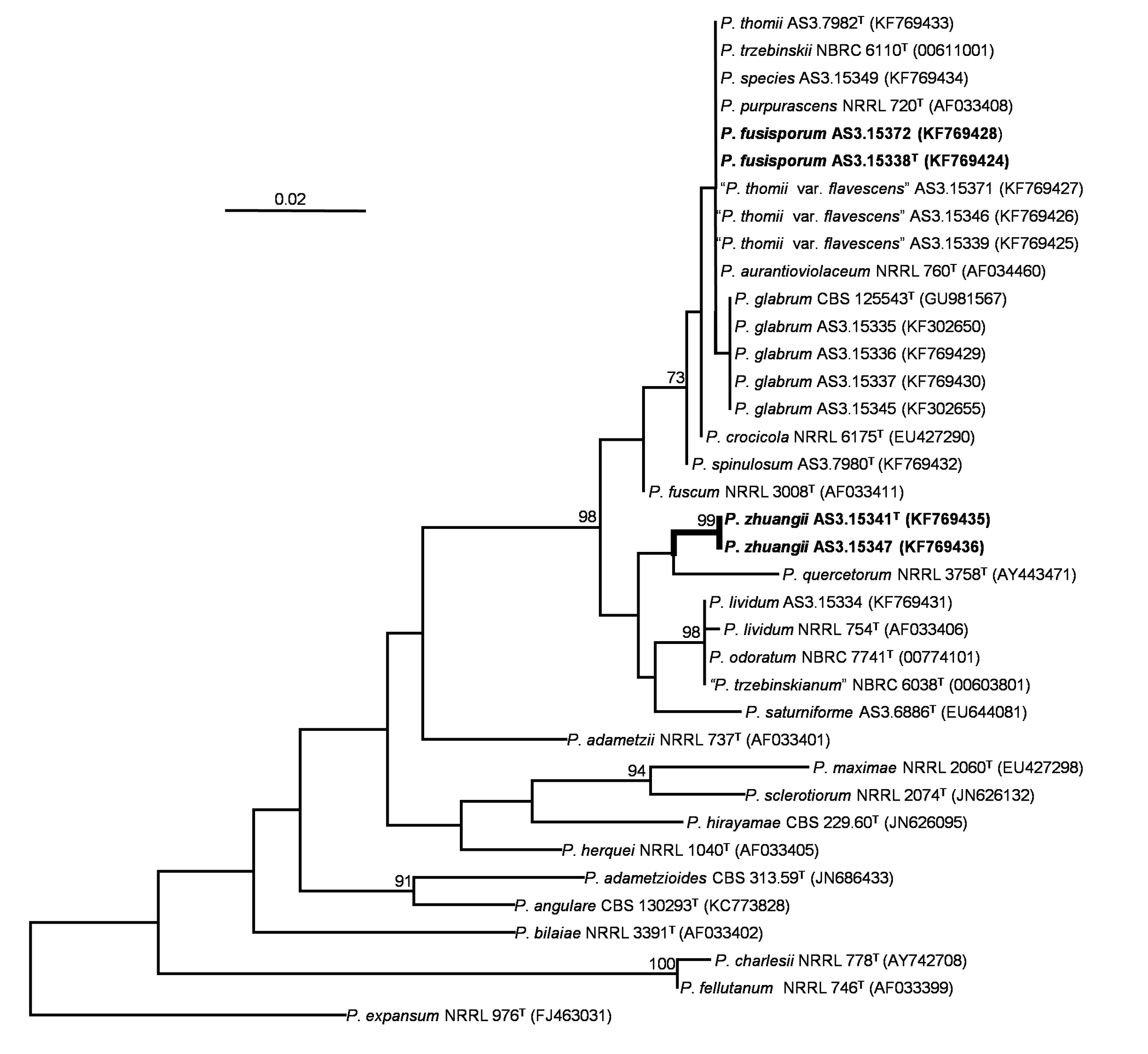

Supplement: Figure S1 — ML phylogram inferred from the ITS1-5.8S-ITS2 sequences. Bootstrap percentages over 70% derived from 1000 replicates are indicated at the nodes. Bar = 0.02 substitutions per nucleotide position. The sequences of P. odoratum NBRC 7741 T, “P. trzebinskianum” NBRC 6038 T and P. trzebinskii NBRC 6110 T were retrieved from the on-line catalogue of Biological Resource Center (NBRC), NITE of Japan (http://www.nbrc.nite.go.jp/e/). (TIF) [file pone.0101454.s001.tif]
